# Reticular design and crystal structure determination of covalent organic frameworks

**DOI:** 10.1039/d1sc00738f

**Published:** 2021-06-07

**Authors:** Ha L. Nguyen

**Affiliations:** Department of Chemistry, UAE University Al-Ain 15551 United Arab Emirates; Joint UAEU–UC Berkeley Laboratories for Materials Innovations, UAE University Al-Ain 15551 United Arab Emirates nguyen.lh@uaeu.ac.ae; Berkeley Global Science Institute Berkeley California 94720 USA nguyen.lh@berkeley.edu

## Abstract

Reticular chemistry of covalent organic frameworks (COFs) deals with the linking of discrete organic molecular building units into extended structures adopting various topologies by strong covalent bonds. The past decade has witnessed a rapid development of COF chemistry in terms of both structural diversity and applications. From the structural perspective, irrespective of our subject of concern with regard to COFs, it is inevitable to take into account the structural aspects of COFs in all dimensions from 1D ribbons to 3D frameworks, for which understanding the concepts of reticular chemistry, based mainly on ‘reticular design’, will seemingly lead to unlimited ways of exploring the exquisiteness of this advanced class of porous, extended, and crystalline materials. A comprehensive discussion and understanding of reticular design, therefore, is of paramount importance so that everyone willing to research on COFs can interpret well and chemically correlate the geometrical structures of this subset of reticular materials and their practical applications. This article lies at the heart of using the conceptual basis of reticular chemistry for designing, modeling, and determination of novel infinite and crystalline structures. Especially, the structure determinations are described by means of chronological advances of discoveries and development of COFs whereby their crystal structures are elucidated by modeling through the topological approach, 3D electron diffraction, single-crystal X-ray diffraction, and powder X-ray diffraction techniques.

## Abstract 2

This article describes the conceptual basis of rational design in COF chemistry and comprehensively discusses the crystal structure determination of COFs using the topological approach, X-ray diffraction, and 3D electron diffraction.

## Graphical abstract

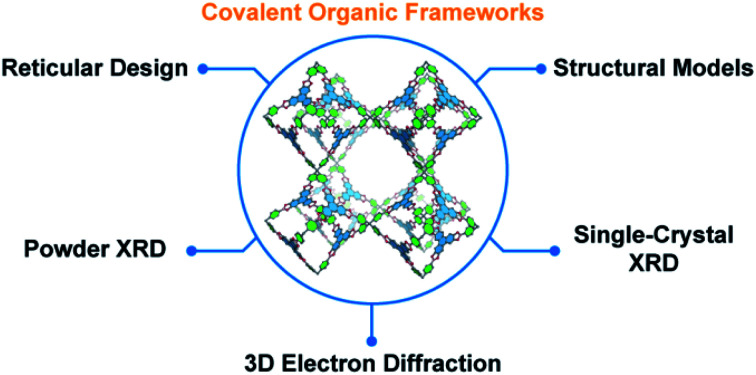

## 1. Introduction

**Fig. 1:**
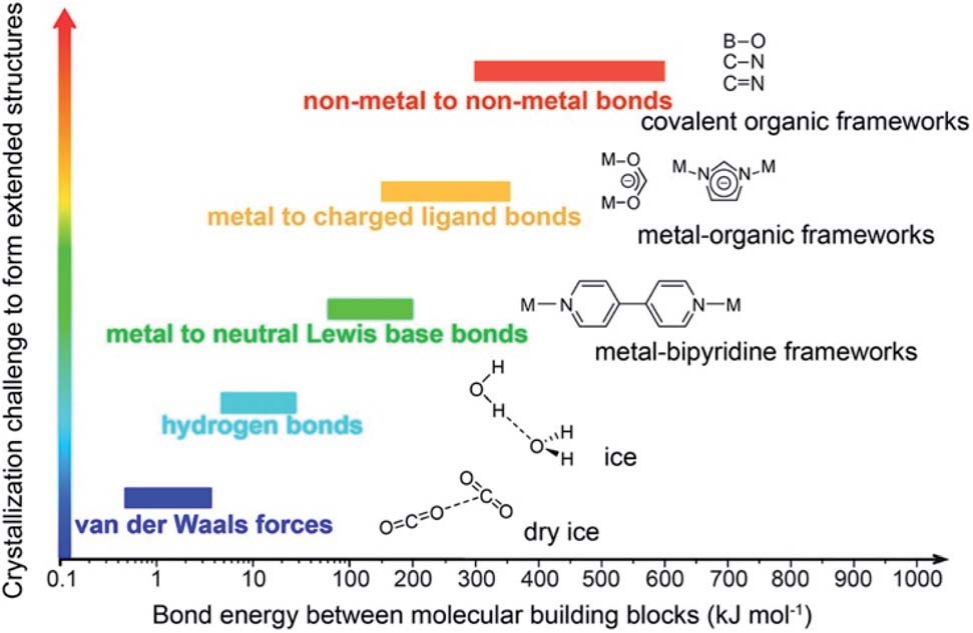
Schematic diagram of the stability and crystallization problem of COFs compared to other extended structures and molecular compounds. Reproduced with permission.^8^ Copyright 2016, American Chemical Society.

Covalent organic frameworks (COFs)^1^ have been drastically developed and their scope has been expanded in both fundamental chemistry and applied science, as evidenced by their research in over one hundred countries after the first landmark report in 2005.^2^ The advanced and groundbreaking traits of COFs rely on the fact that they consist of purely organic linking units crystallizing in the form of porous and extended structures in which bonds building up the frameworks are strong covalent linkages.^3^ Being a subclass of reticular materials, COFs are influenced by and inherit the ability of structural design and modification at the molecular level from reticular chemistry^4^ thanks to their flexibility in the selection of constituents—secondary building units, SBUs. Being extended frameworks linked by strong covalent bonds, COFs are expected to be more stable than their MOF counterparts whose structures are based on metal–oxide links (Fig. 1). Such strong covalent linkages, however, negatively affect the association and dissociation of bonds during the crystallization process, thus reducing the crystallinity of the resulting COFs, which is the biggest challenge faced by the reticular chemistry community.^5,6^ As a consequence, dealing with the crystal structures of COFs is tremendously difficult and becomes the most arduous task. The limitation of the crystallization in COFs not only limits the diversity of COF chemistry but also complicates the structure determination, which is the most important step that must be solved before publishing the research, since it is too challenging to obtain COF crystals with suitable size for structural characterizations. This vexed drawback possibly leads to a severe problem: the mistaken claim of the crystal structures of new COFs, especially when they are formed with novel topologies.^5^ This issue must be avoided.

Due to the exponential growth of reticular chemistry, especially in COFs over the past decade,^2–4^ ‘reticular design’ has been an oft-discussed topic and it inevitably turns out to be a unique concept for designing and engineering COF structures. Particularly, this concept includes rational design of new structures, novel topologies, isoreticular expansion, and post-synthetic modification (PSM).^7^ These aspects are crucial for diversifying the structure library of COFs and further relatively impacting on the chemistry and applications of COFs. In other words, the rich chemistry of COFs relies much on the understanding of reticular design which in turns leads to the best correlation between the crystal structures of COFs and their performances in practical applications to ‘benefit’ the world—the final destination of chemists. Therefore, it is so important that we comprehensively discuss the concept of reticular design on the conceptual basis of reticular chemistry in practice so that (emerging) scholars can capture the big picture of using this idea for designing structures of reticular materials.

Concerning the interplay between COF structures and their chemistry and applications, it is imperative to summarize the conceptual basis and practical guidance of applying reticular chemistry in designing reticular structures and crystallography in COFs. Particularly, this article aims to (1) generally summarize COF chemistry and its advancements, (2) comprehensively discuss the conceptual basis of reticular chemistry for the rational design of new reticular materials, mainly focusing on COFs but which can also be applied to other crystalline materials, and (3) tutorially describe the process and methods of solving the crystal structures of COFs using a variety of techniques. The last objective is largely based on using the topological approach in reticular chemistry to model structures and employing X-ray diffraction (both powder and single-crystal XRD, PXRD and SXRD) and electron diffraction (ED) techniques to determine COF structures.

This article will educate (emerging) scholars on three main lessons: the chemistry of COFs, fundamentals of rational design based on reticular chemistry, and structure determinations from the basics of modeling to advanced techniques.

## 2. Chemistry of covalent organic frameworks

Inorganic porous materials, zeolites, discovered in the eighteenth century, have been largely used as commercial catalysts and adsorbents.^9^ The first porous organic compound reported was Dianin's compound in 1914. This compound intermolecularly formed hydrogen bonds, thus affording empty space for the chemistry of host–guest interaction.^10^ The Dianin compound was called an “organic zeolitic sorbent”. The hydrogen bonds, however, limit 3D structures, weaken the architectural robustness of compounds, and hinder applications related to sorption, separation, and catalysis due to the fragile linkages and backbones.^11^ To circumvent this problem, the fragile hydrogen bonds need to be replaced by strong covalent or coordinative bonds which help rigidly extend the structural frameworks that adopt appealing topological aspects—these cannot be achieved in such materials based on weak bonds.

At this juncture, Yaghi and co-workers inventively developed highly crystalline boroxine- (B_3_O_3_ six-membered ring) and boronic-ester-based (BO_2_C_2_ five-membered ring) COFs, termed COF-1 and COF-5,^12^ respectively. It is worth noting that COF-1 and COF-5 are the first two exemplars whose structures were reticulated from discrete molecules into covalently extended 2D frameworks, adopting permanent porosity and controllable pore sizes. This pioneering work has inspired other studies for developing porous and crystalline COFs with multiple linkages, diverse topologies, and functionallities.

In reticular chemistry, porosity, along with the density of active sites, is a critical factor dictating applications in gas storage, separation, catalytic transformation, and many more. The porosity of COFs is attributed to the cages/pores, formed by reticulating organic SBUs into extended structures; COFs exhibit permanent porosity ranging from hundreds to thousands m^2^ g^−1^. Particularly, DBA-3D-COF-1 displayed the highest surface area in COF chemistry which was reported to be 5083 m^2^ g^−1^.^13^ Along with the record surface area in COFs, the crystal density of DBA-3D-COF-1 was 0.13 g cm^−3^, lower than those of any porous materials reported so far. It should be understood that the density of COFs is much lower than those of other porous materials since COFs are composed of light elements including H, B, C, and N (it, however, does not rule out the fact that COFs can also be made up of other high-density atoms such as halogens, oxygen, or metal elements introduced into the COF structures by PSM). Unlike other amorphous materials (*e.g.*, activated carbon, amorphous polymers), COFs crystallize from various organic SBUs by co-condensation, self-condensation, or ring-closing reaction and most importantly, COF structures can be determined by X-ray diffraction (XRD), thus allowing the structural optimization at the molecular level.^14^ The crystallinity of COFs relies much on the dynamically reversible bonds being capable of self-correcting the errors that occur during synthetic processes which can be varied and controlled. Especially, COFs with high crystallinity have minimal defects making them attractive in many studies ranging from atomic to macroscopic level-based devices. This feature, the well-defined structure and high crystallinity, distinguishes COFs from amorphous polymers in many applications related to the foundational understanding of molecular interaction, structure tailoring, PSM, and synergic effects.^15^

In terms of structural constituents, COF structures are based on linkages inherited from organic chemistry including reversible (boroxine,^12^ boronic ester,^12^ imine,^16^ borosilicate,^17^ spiroborate,^18^ triazine,^19^ azine,^20^ imide,^21^ squaraine,^22^ hydrazone,^23^ urea,^24^ and olefin^25^) and irreversible (β-ketoenamine,^26^ amide,^27^ benzoxazole,^28^ imidazole,^29^ and dioxin^30,31^) links. Additionally, linkages formed by nucleophilic aromatic substitution (S_N_Ar), by virtue of the dynamic and self-correcting nature, generate extended structures with large surface area and high chemical stability.^32^ Exploring COFs made up of such stable linkages is essential for practical applications.^30,31^

As mentioned above, the reversible-link chemistry permits self-correction during the synthetic process, hence, enhancing the crystallinity which is an important factor determining the optimized porosity and minimizing defects. Such COFs based on reversible links, however, suffer from hydrophilic reversibility which makes them unstable under harsh conditions (aqueous, acidic, or basic medium) and limits their practical applications. This issue can be circumvented by (1) engineering the internal structure of COFs,^26^ (2) replacing the reversible linkages with irreversible ones^33^ (although using the irreversible links requires appropriate synthons to obtain COFs with high crystallinity), and/or (3) postsynthetically modifying linkages (linkage conversion).^34^

The stability of COFs is determined by how long the frameworks can exist under the tested conditions which depends mainly on the strength of linkages creating the MOF structures. Owing to the covalent bond nature, the COF stability is expected to exceed that of the MOF counterparts. Particularly, COFs based on robust covalent bonds such as β-ketoenamine, amide, and benzoxazole are strongly water-stable as well as acid/base resistant. It is worth noting that the improvement of chemical stability in COF chemistry has sparked the most attention in which COFs with new bond-formation have been pursued recently. Apart from the strength of linkages, other factors including the void space of the structure, hydrophobic functionalities, intramolecular conjugation, and/or structural accessibility might contribute to the structural rigidity and stability of COFs.^35^

## 3. Reticular design in covalent organic frameworks

Topologies or nets were first described by Wells^36^ since the use of SXRD for the structural determination and visualization of crystalline materials. This definition of topology or structure type uses the concepts of vertices and edges where the atoms, considered as vertices, are linked together by chemical bonds (edges) to generate periodic and infinitive graphs. In other words, topology considers all symmetry operations under the graph theory (*i.e.*, topology permits a simplified structure to be stretched, compressed, bent, and flexed without tearing or destroying the geometrical connectivity). Chemists use topology as the specific fingerprint to (1) differentiate structures constructed from similar shapes of SBUs and (2) rationally design crystalline materials.^37,38^ Particularly, databases (*e.g.*, Reticular Chemistry Structure Resource (RCSR), ToposPro, or Systre)^37,39^ were developed to categorize the vast number of networks in chemistry. In particular, Yaghi and co-workers developed the use of topological symbols consisting of three-letter symbols, lowercase and bold, in the early 2000s, which are widely used in the reticular chemistry community.^39^

The rational design of COF structures in light of the basic concept of reticular chemistry is described as follows. First, a specific topology, whose connectivity of vertices and edges and topological information (*e.g.*, natural tiling, dual, face) can be found in detail in RCSR, is targeted. Second, based on the detailed information of the topological structure from RCSR, the kinds of vertices and edges are identified. As a result, the geometrical shape of building units akin to the chemical building blocks used for constructing COFs will be realized and appropriately selected. Finally, such chemical equivalents are chemically reticulated into the extended COF structures. This conceptual basis works well with an array of highly symmetric topologies and dictates the formation of targeted structures, allowing structural design in a rational manner.

In order to exemplify this basic concept of reticular design, we discuss two examples: the first example is COF-5 with a honeycomb (**hcb**) topology^12^ and the second one is COF-102 and COF-108,^40^ the first reported 3D COFs with **ctn** and **bor** topology, respectively. Indeed, the **hcb** network consists of one kind of vertex which are triangles tethered by linear building units.

**Fig. 2:**
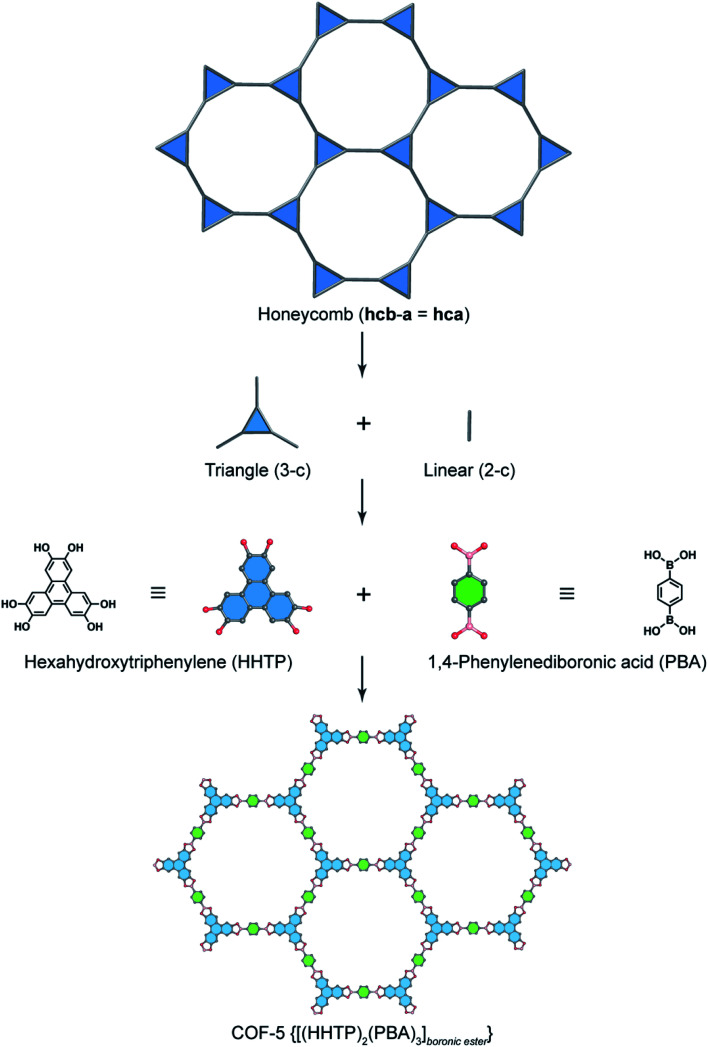
Reticular design of honeycomb topology. Deconstruction of the augmented net of honeycomb (**hcb-a**, also termed **hca**) into triangular (3-c) and linear (2-c) units which correspond to hexahydrotriphenylene (HHTP) and 1,4-phenylenediboronic acid (PBA), respectively. HHTP and PBA are reticulated into the 2D structure of COF-5 [(HHTP)_2_(PBA)_3_]_boronic ester_ adopting boronic ester linkages.

**Fig. 3:**
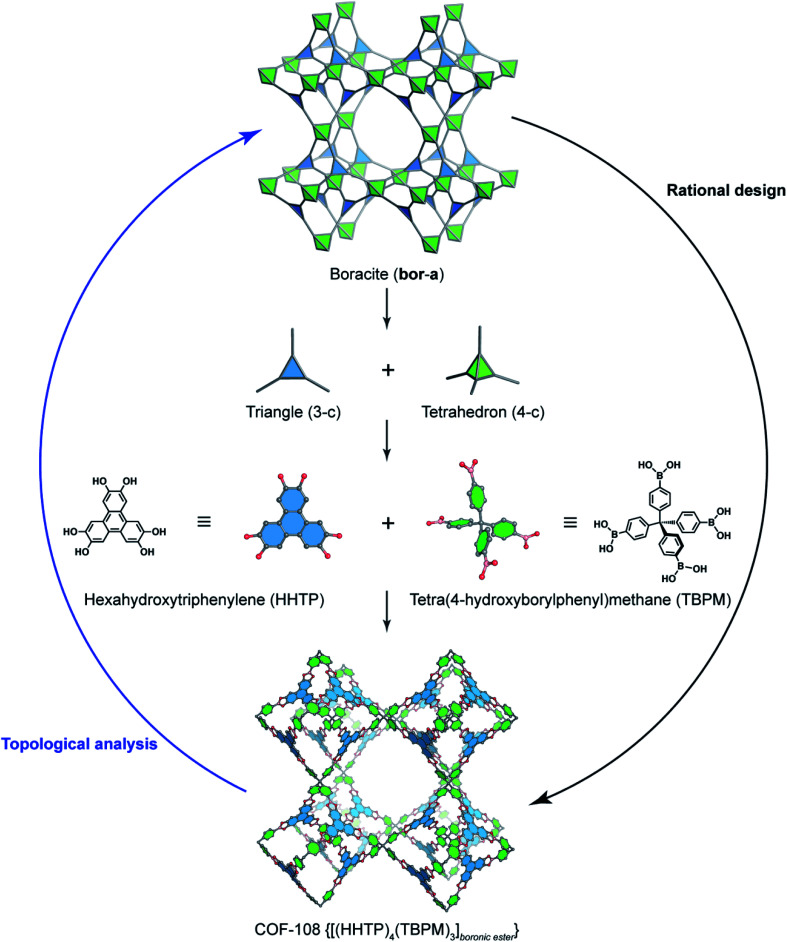
Reticular design of the augmented boracite topology (**bor-a**) which is deconstructed into triangles (3-c) and tetrahedra (4-c) corresponding to HHTP and tetra(4-hydroxyborylphenyl)methane (TBPM), respectively. TBPM reacts with HHTP to form COF-108 [(HHTP)_4_(TBPM)_3_]_boronic ester_. The opposite way of rational design is the topological analysis.

Deconstructing the **hcb** topology (Fig. 2) leads to the requirement of a triangular linker and ditopic linker corresponding to hexahydroxytriphenylene (HHTP) and diboronic acid (BDBA), respectively. The condensation reaction, in a stoichiometric ratio, between HHTP (one mole) and BDBA (1.5 mole) leads to the formation of COF-5 crystallizing in a layer-by-layer structure adopting hexagonal pores as expected. Using a similar topological deconstruction, we can rationalize the formation of COF-108 possessing the **bor** topology (Fig. 3). Particularly, **bor** comprises triangles and tetrahedra formed in such a way that the structure is composed of two distinct cages: octahedral and cuboctahedral shape. Taking this into account, Yaghi and co-workers reticulated hexahydroxytriphenylene (HHTP; 3-c) and tetra(4-hydroxyborylphenyl)methane (TBPM; 4-c) into [(HHTP)_4_(TBPM)_3_]_boronic ester_ (COF-108) through a co-condensation reaction that formed boronic ester linkages, similar to COF-5. In another reaction, the self-condensation of TBPM units led to the formation of COF-102 adopting the **ctn** topology with boroxine linkages.

One may ask “how can we know the exact topology formed when combining two SBUs?” Indeed, we were asked this question by our colleagues and students; we also believe that this question will be frequently asked by emerging scholars working on reticular chemistry. In order to comprehensively discuss this question, it is worth paraphrasing it from the original form to “what are the ways of reticulating organic SBUs based on triangles, squares, tetrahedra, triangular prisms, *etc.* into extended frameworks?” The answer is: there are many ways. Nevertheless, there are some topologies that can mostly be targeted. Such topologies fall under the umbrella of ‘reticular synthesis’. To this end, one kind of link (*i.e.*, one kind of vertex) must be used. Interestingly, this leads to the next question which is “how many such topologies?” The answer is “there are not many possibilities and they are the main targets that reticular chemistry is dealing with using reticular design”.

**Fig. 4:**
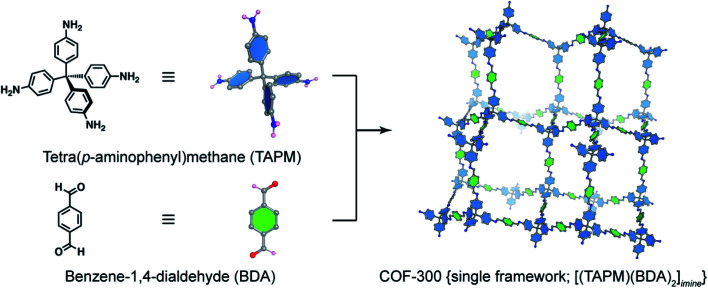
Combination of a tetrahedral unit, tetra(*p*-aminophenyl)methane (TAPM), and a linear linker, benzene-1,4-dialdehyde (BDA), generates a 5-fold interpenetrated framework of COF-300 {[(TAPM) (BDA)_2_]_*imine*_} with diamond topology (**dia**). Only a single framework of COF-300 is displayed for clarity.

The basic concept of reticular chemistry has been largely employed and COF chemists indeed have reported notable achievements.^41^ For example, the chemistry of imine-linked COFs was pervasive and drastically explored since the first report of COF-300 (ref. 16) with a diamond (**dia**) topology whereby the amine-functionalized tetrahedral units, tetra(*p*-aminophenyl)methane (TAPM), connect together through linear benzene-1,4-dialdehyde (BDA) linkers (Fig. 4). Multiple frameworks of COF-300 tended to grow at the same time during the crystallization to avoid the huge void space of the single framework, thus generating a 5-fold interpenetrated structure (the topology is denoted as **dia-c5**). It is accepted that the interpenetration maximizes the stability of the resultant material and its crystallinity.

From the topological perspective, if we start using the specific linker based on the tetrahedral geometry, there are several ways to reticulate them into 3D frameworks using 2-, 3-, 4-, to 6-, or even 8-coordinate SBUs (2-c, 3-c, 4-c, 6-c, and 8-c). Particularly, linear linkers connect the tetrahedral building units to generate a **dia** network.^42^ Triangular linkers link the tetrahedral SBUs to form **ctn** or **bor**^40,43^ where the formation of **ctn** or **bor** depends mainly on the geometrical hindrance of the triangles.

Increasing points of extension of SBUs to 4 (4-c + 4-c) leads to two topological possibilities to obtain different kinds of structures. If 4-c + 4-c is tetrahedral SBU + another tetrahedral SBU, **dia** or **lon**^44^ will be generated; if 4-c + 4-c is the tetrahedral SBU + square SBU, **pts**^45^ or **pth** will be formed. Indeed, an extended 2-fold interpenetrated structure of 3D-Py-COF adopting the **pts** network was first reported by Wang and co-workers using the combination of the tetrahedral building unit of tetra(*p*-aminophenyl)methane (TAPM) and square-planar unit of 1,3,6,8-tetrakis(4-formylphenyl)pyrene (TFPPy).^45^ Unfortunately, neither COFs based on **pth** nor other novel topologies that are reticulated from 4-c (tetrahedral unit) and 6-c (hexagonal or triangular prism unit) or 4-c and 8-c (cube) have been reported. Very recently, the approach of using 6-c SBUs for novel 3D COF synthesis has been achieved to some extent. Particularly, Cooper and coworkers reported the synthesis of the **acs** topology COF based on triangle prism (6-c) and linear (2-c) building units.^46^ Later, this 6-c SBU was linked by 3-c and 4-c to form **ceq**^47^ and **stp**^48^ topologies, respectively.

The chemistry of 3D COFs still provides many opportunities to those who are willing to take challenges because increasing the valency^49^ of organic SBUs mostly requires tedious synthetic procedures.

All the aforementioned 3D networks of COFs are uninodal (**dia**, **lon**) whose structures were built with one kind of vertex and binodal (**bor**, **ctn**, **pts**) where the structures were composed of two kinds of vertices. To increase the complexity of 3D COF chemistry, Feng and co-workers reported a 3D anionic cyclodextrin-based COF (CD-COF) with a trinodal network of **rra**.^50^ Particularly, a six-membered ring of γ-CD simplified by (3,3)-c and trimethyl borate (B(OMe)_3_) were co-condensed to generate the trinodal (3,3,4)-c **rra** network. This strategy showcased the fact that increasing the complexity in COFs opens a new pathway for overcoming the challenges of 3D COF synthesis. To this end and to apply suitable methods for directing the connectivity in combining triangles and squares to create COFs with 3D topologies, we have recently reported the conformational design that relies on modifying the dihedral angles,^51^ between 74 and 90°, by introducing methyl groups to the central phenyl ring of the triangular SBU which thereby selectively generate the 3D COFs based on the **fjh** topology. Interestingly, the linker lacking conformational design only reacts with square-planar SBUs to form amorphous products. This work relying on the predesigned SBUs paves the way for the designed synthesis of novel 3D COFs by modifying the geometrical structures of starting materials. Very recently, by using a similar strategy, Sun and Wang *et al.* reported topology control in 3D COFs.^52^ In particular, by tuning the geometry of building units using steric hindrance, 3D COFs including 5-fold interpenetrated **pts** (methoxy was introduced on the square unit) and 2-fold interpenetrated **ljh** (phenyl was substituted on the square unit) were obtained.

It is beyond doubt that comprehensive understanding of topology is imperative for the designed synthesis of novel COFs with a variety of structure types and is a key factor for the success of COF chemistry. The structural designs and discoveries of COFs having diverse topologies are based on three main concepts: geometry of linking units, geometrical constraints, and frustrated chemistry. In terms of targeted nets constructed through the conceptual basis of reticular chemistry, building-unit geometry plays an essential role, as presented early in this section (*e.g.* , **hcb**, **bor**, **dia**, **pts**, **rra**, *etc.*). Regarding the selective crystallization of specific topologies, geometrical conformation of SBUs is the most effective method to rely on, as recently proven by a few examples (**fjh** and **ljh**). Last but not least, frustrated chemistry provides a powerful strategy to achieve cutting-edge designs for synthesizing COFs bearing unique structural topologies (ribbon-like net and defective **tth**^53^). Table 1 summarizes the topological features of 3D COFs, which may help provide useful information on how 3D COFs can be made up of various building units. It is clear that 3D COFs constructed by the combination of 4-c and 8-c have not been reported yet. Along with this, COF topologies with high connectivity (larger than 8-c) are muted.

**Table 1:** Summary of topologies in 3D COFs

Connectivity	Vertex geometry	RCSR symbol	Kinds of vertices and edges
3	Triangle	**rsr**	11 (uninodal)
4	Tetrahedron	**dia**	11 (uninodal)
4	Tetrahedron	**lon**	12 (uninodal)
6	Triangular prism	**acs**	11 (uninodal)
8	Cube	**bcu**	11 (uninodal)
3,4	Triangle, tetrahedron	**bor**	21 (binodal)
3,4	Triangle, tetrahedron	**ctn**	21 (binodal)
3,4	Triangle, square	**tbo**	21 (binodal)
4,4	Tetrahedron, square	**pts**	21 (binodal)
4,6	Square, triangular prism	**stp**	21 (binodal)
3,3,4	Triangle, square	**rra**	33 (trinodal)
3,4,4	Triangle, square	**ffc**	32 (trinodal)
3,4,4	Triangle, square	**fjh**	32 (trinodal)
4,4,4	Tetrahedron, square	**ljh**	32 (trinodal)
3,6,6	Triangle, triangular prism	**ceq**	32 (trinodal)

## 4. Crystallography in covalent organic frameworks

Determining the single-crystal structures of crystalline compounds elucidates the interplay between the chemical structures and their physicochemical properties, and therefore their applicability. Especially, in the reticular chemistry of COFs, structural determination is the most important and key factor to explain and further cast light on COFs' outstanding behaviors in various applications (gas storage, water uptake, catalysis, or conductivity).^54^ PSM based on the structural comprehension then becomes accessible.^55^ Nevertheless, owing to the crystallization challenge, COFs are normally obtained with low crystallinity (*i.e.*, small crystal size and therefore less PXRD peaks) which hinders the elucidation of their crystal structures by both ED and XRD techniques.^44^ To date, solving the crystal structures of COFs is one of the major challenges faced by scientists working on reticular chemistry. This section, therefore, aims to (1) overview fundamentals of the structural elucidation in COFs, (2) highlight the recent achievements on this topic, and (3) educate (emerging) scholars on how to determine the crystal structures of COFs.

### 4.1. Structural models based on reticular chemistry

It is worth clarifying that the COF crystallography has been relying on the structural modeling for a long time starting from the discovery of 2D and 3D COFs by Yaghi and colleagues.^12,40^ Indeed, the modeling of COFs lies in the conceptual basis of reticular chemistry whereby the reticulation of various SBUs into extended structures is predictable. The structural modeling proceeds through a heuristic approach including searching for possibilities, eliminating results, and matching possible models. At the end, the possible model structures are refined using the Pawley or Le Bail method. It is worth noting that this modeling process acting as a standard for reporting COF structures was put forth over a decade ago and is still applicable to many cases in which the crystallinity of COFs cannot be improved due to the crystallization challenge.

**Fig. 5:**
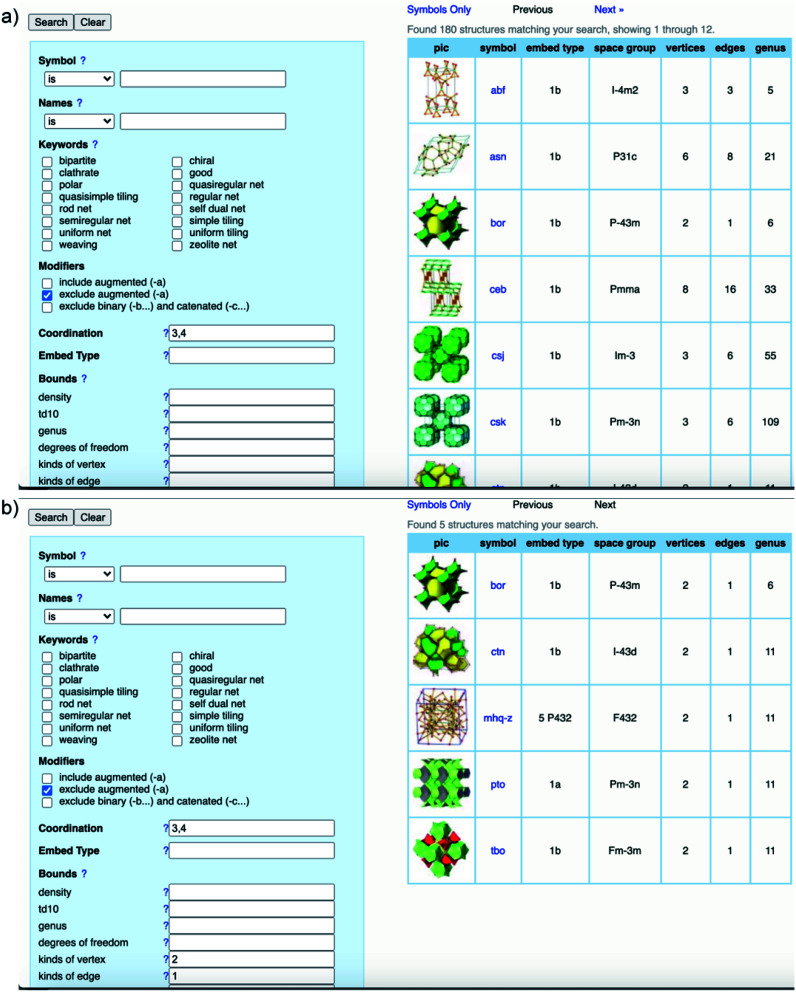
Heuristic process of finding **bor** and **ctn** topologies through the RCSR database. General search using 3,4-coordinate SBUs results in 180 possible topologies (a) and minimizing the possibilities by introducing kinds of vertices and edges (b). The search was conducted on November 10, 2020.

To comprehend the detailed process of the heuristic approach using reticular design, the structural modeling of the first 3D COFs (COF-102, COF-103, COF-105, and COF-108)^40^ is described using the following steps (Fig. 5). The TBPM linker or its analog tetra(4-dihydroxyborylphenyl)silane (TBPS) and the triangular linker (HHTP) were chemically reacted to form crystalline products. The tetrahedral and triangle links are connected through appropriate linkages. In the next step, several possible topologies can be found using the search function from the RCSR database. In the search box, 3 and 4 (typing 3,4) are introduced as coordination representing the combination of 3-c and 4-c. To minimize the possibilities, augmented nets (the augmented net is formed by replacing the vertices of the original net with the geometrical shape of SBUs—the group of vertices) should be excluded. With this input, 180 possible networks are obtained. Because the highest symmetry is preferred, the combination of tetrahedral and triangular linkers will generate two kinds of vertices and one kind of edge. The kind of vertices and edges will then be specified in the search box to finally reduce the topological possibilities from 180 to 5: **bor** (boracite), **ctn** (cubic-C_3_N_4_), **mhq-z**, **pto** (Pt_3_O_4_), and **tbo** (twisted boracite). The suitable topologies are **bor** for COF-102, COF-103, and COF-105 and **ctn** for COF-108 whose structures comprise tetrahedra and triangles while the latter three structure types (**mhq-z**, **pto**, and **tbo**) are constructed from squares and triangles. The last step is the structural modeling and the PXRD refinement of the structural models against the experimental PXRD patterns. As mentioned above, Pawley and Le Bail fittings are often used. This heuristic process will be able to accurately predict the most preferred structures formed by various kinds of SBUs without considering linkages or structural features including pore size, empty space, density, and surface area. Additionally, this top-down approach plays an essential role in discovering new COF structures. Especially, this works well with the targets of reticular chemistry—structures resulting from linking one kind of vertex and one kind of edge.^46^

Albeit powerful and irreplaceable, the heuristic method remains challenging and has limitations: it is most applicable to high symmetric SBUs and the results suggest only reported topologies. This means scientists will have to play around with the modeling with enormous efforts to simulate the structures and go through the trial and error process. Moreover, this method falls short of predicting the novel topologies of COF structures which must be first determined by the XRD or ED technique. The final drawback of the heuristic approach is structural refinement. Let us be clear at this point: a model structure can only be validated by Rietveld refinement^56^ but not the others (Pawley or Le Bail). Many scholars misuse, even misunderstand, these terminologies. Pawley and Le Bail refinements do not provide the structural coordination information except for the convergence of the unit cell parameters. Therefore, it is mandatory to corroborate the models by additional characterizations that show the stoichiometric ratio of linkers, linkage formation, pore size distribution, theory of surface area (if applicable), elemental compositions and ratios, and as many as possible from this list. In this context, we would like to direct the readers to the critical review of standard practices in reticular chemistry reported by Yaghi and colleagues.^2^

### 4.2. Structure determinations by 3D electron diffraction

Electron diffraction was first reported in 1927 by Thomson and Reid.^57^ Unlike X-rays or neutrons used in diffraction to study structures of materials, electron diffraction is based on charged particles that interact with crystalline materials through Coulomb forces.^58^ That is the reason why both positively charged atomic nucleus and negatively charged electrons are influenced by the incident electron beam, thereby resulting in strong scattering of electrons which was not considered for the application of structural determination of crystalline materials until 2007 when semi-automated techniques for ED-data collection were apparently developed.^59^

The advances of ED lie at the heart of two main factors: first, the possibility to collect electron diffraction data of samples that crystallize in the size of nanometers, an order of magnitude smaller than crystals that are used for single-crystal analysis using either a laboratory diffractometer or synchrotron irradiation; second, diffraction and imaging data can be combined in ED measurements which permit the correlation between 3D reciprocal space and direct method for phase identification and structural solution similar to that of SXRD analysis.^60^

ED measurements require expertise in handling the sample, setting up experiments, and data acquisition. The past decade has witnessed the rapid development of programs for reconstruction and virtualization of reflections collected by ED into a 3D reciprocal space. Details of the historical development of ED have been recently discussed in the literature.^60,61^ To date, ED techniques applied for COF structure determinations include two common methods for collecting data: rotation electron diffraction (RED) and continuous rotation electron diffraction (cRED). Zhou and Huang at Stockholm University and Terasaki and Ma at ShanghaiTech University are widely known for their expertise in solving crystal structures of reticular materials using the ED techniques.

**Fig. 6:**
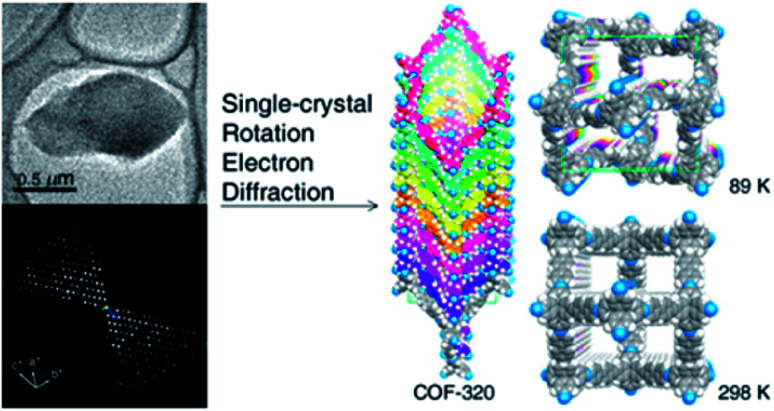
Single-crystal structure analysis of COF-320 using the RED technique. COF-320 crystallized in single sub-μm crystals (top left) and was the first sample for which RED data were collected and reconstructed to the 3D reciprocal lattice (bottom left). Right side shows the crystal structure of the 9-fold interpenetrated **dia** framework solved at 89 K and 298 K. Reproduced with permission.^62^ Copyright 2013, American Chemical Society.

The first achievement in solving the single-crystal structure of COFs was COF-320 (Fig. 6)^62^ composed of tetra(4-anilyl)methane (TAM) and 4,4′-biphenyldialdehyde (BPDA). The combination of these units, based on reticular chemistry, would generate a structure with the diamond (**dia**) topology. COF-320 was measured using RED at both 298 and 89 K, wherein the latter temperature reduced the structural damage by the electron beam. Data collection for COF-320 was carried out best at 89 K using the following procedure: the COF was tilted in the range of −34.19–38.33° with a step width of 0.20°. The total measured time was 21 m; 396 ED frames were obtained which corresponded to 570 unique reflections after the reciprocal reconstruction. COF-320 can be measured with a resolution of up to 1.5 Å. Simulated annealing was applied to locate the positions of TAM and BPDA building units. Subsequently, the crystal structure of COF-320 was refined against the RED data to reveal a 9-fold interpenetration of the **dia**-based framework. At 298 K, only central C atoms of TAM building blocks were located. Therefore, modeling was built and then refined against the PXRD pattern to finally lead to a similar structure to COF-320 solved at 89 K, except for disordered N atoms.

**Fig. 7:**
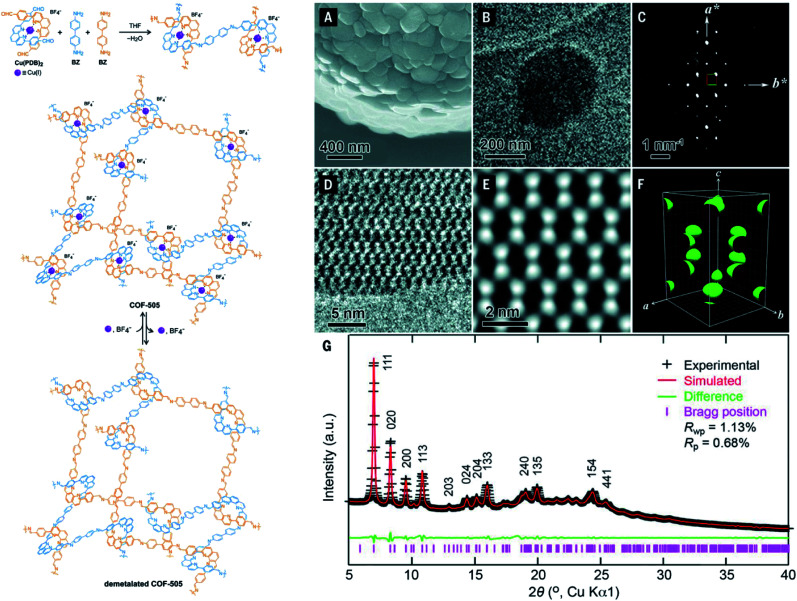
Schematic reaction for synthesis of the woven structure, COF-505 (left). Characterizations of morphology and electron microscopy of COF-505 (right): (A) SEM image of COF-505. (B) TEM image of COF-505. (C) Reconstructed reciprocal lattice of COF-505 collected at 298 K from a set of 3D EDT (3D electron diffraction tomography) data. (D) HRTEM image of COF-505 along the 1–10 lattice plane. (E) Imposing *pgg* plane group symmetry on (D) leads to the 2D projected potential map of COF-505. (F) The 3D electrostatic potential map of COF-505 shows positions of Cu atoms. (G) PXRD pattern analysis of the activated COF-505 through Pawley refinement. Reproduced with permission.^63^ Copyright 2016, American Association for the Advancement of Science.

It is apparent that nanometer-sized crystals (a few hundreds) are possibly suitable for ED measurements with a short collection time. The resolution of ED measurements, however, is still low and it is not efficient to fully solve the crystal structures of COFs without other aided methods such as high-resolution transmission electron microscopy (HRTEM), modeling, and Rietveld refinement (the latter two methods will be discussed in Section 4.4). This is proven in the case of COF-505-Cu^63^ whose structure consists of woven building units (copper(i)-bisphenanthroline tetrafluoroborate, Cu(PDB)_2_(BF_4_)) linked by linear amino-functionalized linkers (benzidine (BZ)) (Fig. 7). Particularly, unit cell parameters and reciprocal lattice were obtained which suggested several space groups. The final space group was firmly confirmed by HRTEM images analyzed along the 1–10 lattice plane. Importantly, the position of Cu(i) atoms was located by Fourier analysis of HRTEM images, thus allowing the structure of COF-505-Cu to be modeled. The Pawley method was used again to refine the unit cell parameters of COF-505-Cu.

Unlike COF-505-Cu, a series of 3D-TPB-COF-X (TPB = 1,2,4,5-tetraphenylbenzene; X = H, Me, and F, respectively) could be synthesized with a larger crystal size (*ca.* 1 μm) and diffracted at much higher resolution (0.9–1.0 Å).^64^ In this work, for the first time, the crystal structures of three isoreticular COFs, based on the combination of TPB-functionalized aldehyde (square unit) and TAPM (tetrahedral unit), could be fully resolved by cRED. The high-resolution of these 3D **pts** COFs was probably attributed to the high level of interpenetration (5-fold). At the same time, Zhang and Ma *et al.* published a study using the cryo-EDT technique to solve the crystal structure of COF-300 crystallizing with submicrometer sizes.^65^ More importantly, the structural dynamics observed when molecular guests (*e.g.*, water, ionic liquid, and poly(methyl methacrylate)) are incorporated in the pores of COF-300 could be fully determined and revealed at the atomic level with a resolution of 1.1 Å.

It is worth noting that the crystal structure elucidation in COF chemistry is a daunting task even using 3D ED measurements, which is due to the poor crystallinity of COFs, aggregated crystals, and the electron beam damage of COF crystals.^65^ In order to solve this problem, various effective methods for COF crystal structure determination are required. Bearing this in mind, Ma, Zhang, and Harris *et al.* used the direct-space strategy to solve the crystal structures of COF-300-activated and -hydrated forms.^66^ Especially, in this strategy, the authors were able to determine the crystal structure of COF-300 even with a low resolution of 3.78 Å obtained by 3D ED thanks to the direct-space genetic algorithm which, as mentioned by the authors, “effectively explores the *R*-factor hypersurface by mimicking the processes of biological evolution”.^66^ This work showcased its potential to elucidate crystal structures of COFs crystallizing with submicrometer sizes. Impressively, it can be applied to elucidate the crystal structures of COFs even at low-resolution 3D ED data.

Although the ED technique has been rapidly developed over the past decade and contributes to solving many crystal structures of nanometer-sized crystals including MOFs and COFs,^67,68^ it remains challenging.^60^ First of all, most of the COF structures solved by ED analysis either contain heavy atoms^63^ in the form of metalated building blocks or adopt multi-fold interpenetration,^62^ both of which would increase the resolution of ED measurements. The question is whether the ED technique works well with COFs, especially 3D COFs, whose structures are not interpenetrated (*i.e.*, single-framework structures). Secondly, the resolution of ED in such measurements is still quite low. Therefore, it is mandatory to combine ED with other supporting characterizations^69^ (*e.g.*, HRTEM, nitrogen isotherms, *etc.*) to determine COF structures. At this point, combining the aforementioned methods and structural solution by PXRD (see Section 4.4) is worth pursuing. To this end, the COF must be crystallized with a crystallinity as high as possible so that the overlapping problem of the PXRD pattern can be minimized.

### 4.3. Structure determinations by single-crystal X-ray diffraction

**Fig. 8:**
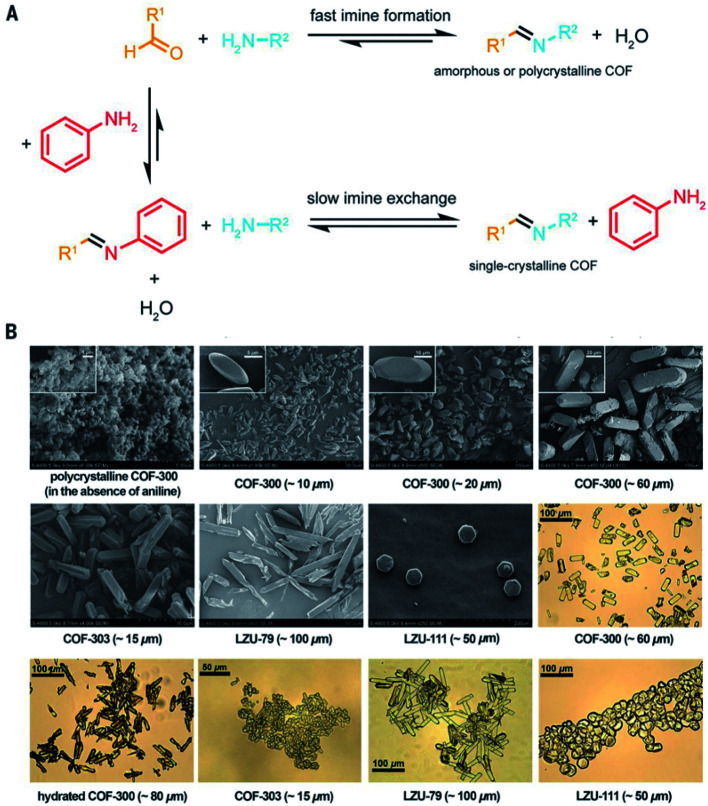
Comparison between fast imine condensation (in the absence of aniline) and slow imine condensation (in the presence of aniline) reaction (A). The slow imine exchange enables the formation of single crystals of 3D imine-based COFs (B). Reproduced with permission.^44^ Copyright 2018, American Association for the Advancement of Science.

It is beyond doubt that the SXRD measurements can resolve the crystal structures of COFs wherein lattice parameters, atom positions, connectivity, and structural information are precisely unveiled. The structural modeling along with PXRD refinement using the Pawley method, as mentioned above, takes a long time and has been used as a standard to report new COF structures until 2018 when 3D imine-linked COFs, for the first time, were crystallized in single crystals.^44^ Particularly, single crystals of COF-300, a hydrated form of COF-300, COF-303, LZU-79, and LZU-111 can be grown as large as 10–100 μm, ideally suited for SXRD measurements using a laboratory diffractometer. The idea of this work lies in the use of nucleophilic aniline as a modulator to first react with the aldehyde starting material to form the model compound which will then be substituted by the ditopic amino-functionalized linker through linker exchange to finally generate the extended framework (Fig. 8). Due to an excess amount of aniline used in the synthesis and the requirement of a slow reaction rate, the reactions took place at room temperature for at least 30 d. This rate-controlled reaction ensures that the single crystals are formed with high quality and therefore the resolution of SXRD measurements reaches up to 0.83 Å. This breakthrough report offers an opportunity to further study the interplay between adsorbates and the COF framework: the host–guest interaction. Indeed, the hydrogen bonds are formed by water molecules and the imine-linked backbone of the hydrated COF-303. This explains somewhat how several imine-based COFs show high water uptake capacity.^70^

Sun and co-workers then continued applying this powerful method along with an aging process to study the interpenetration isomerism of COF-300.^71^

These studies, however, draw our attention to another issue which is the generalizability—the modulator approach up to this point works well only for 3D imine-linked COFs.^72,73^ Recently, Yaghi and co-workers have reported single crystals of a 2D BP-COF-6 based on boron–phosphorous linkages;^49^ it is likely that the single crystals of BP-COF-6 are *in situ* formed under acidic conditions and are not applicable to the vast chemistry of COFs based on a variety of linkages (though the formation of BP-COF-6 paves the way for future use of specific linkers that are able to generate organic clusters *in situ*). At this point, it should be noted that COF chemists are still facing a great challenge with the limitation of COF crystallization, and new chemistry with impactful solutions is highly needed to open the avenue for diversifying COF chemistry.

### 4.4. Structure determination by powder X-ray diffraction

Due to the thermodynamics of organic reactions forming COFs whereby the covalent bonds are stronger than the metal–oxide bonds in MOFs, the bonding association and dissociation in COFs are more unfavorable than in the MOF counterparts, and therefore suffer from kinetic hindrance. As a consequence, COFs usually crystallize in the form of micro-crystalline products, instead of single crystals, and hence are not applicable to the SXRD analysis. The ED analysis has been proven to be useful for determining COF structures when COFs crystallize in micron particle size; this technique, however, suffers from a limitation which is low resolution thereby leading to the failure of the *ab initio* structure solution.^69^

The tendency to obtain microcrystalline COFs along with the low resolution of ED data makes the structural elucidation tedious and laborious, or even impossible in many cases.

The question is what should we do if the structural models following Section 4.1 do not match with the experimental results (*i.e.*, the simulated PXRD patterns are different from the experimental one). That also means how we solve a new COF structure using PXRD data. This section seeks to provide guidance on how we elucidate a new COF structure by its PXRD pattern—this useful but sophisticated technique can also be applied to other crystalline materials.

Solving the crystal structures using PXRD data dates historically back to the early 1990s.^74–78^ During the prosperous developmental stage of zeolite chemistry, McCusker and co-workers developed a method to solve the crystal structure of a complex zeolite (UTD-1)^79^ containing a large number of nonhydrogen atoms (69 atoms in the asymmetric unit); the method relies on using a textured sample and extraction of high intensity of XRD data from synchrotron radiation.

In principle, the structure determination by PXRD goes through three steps: (1) indexing of the PXRD pattern, (2) integration of intensity, (3) structure solution, and (4) refining the structure by Rietveld refinement and only by this refinement. Particularly, powder-pattern indexing executed through standalone programs suggests the possible unit cell and lattice parameters. In the second step, calculation of integrated intensities of diffraction peaks of the powder pattern provides the experimental |*F*_*hkl*_| structure modulus corresponding to the structural phases (eqn (1)). The electron density directly proportional to the inverse of the structure factors gives us atom positions of the structure. In general, reflections of a single crystal at specific diffraction angles are separated and accurately contain information of intensities (eqn (2)). In the diffraction pattern of a polycrystalline material, such reflections, however, overlap with the others owing to the nature of random distributions of polycrystals. Therefore, the intensity integration of crystalline powders falls short of the required information for the conventional approach of structure solution. In order to maximize the resolution of structure determinations, especially by using PXRD, it is essential to collect as many diffraction spots as possible (*i.e.*, integrated intensities are optimized). This is a key factor that determines the success in powder solution by the direct method in step (3) (*ab initio* solution). The higher the crystallinity, the greater the opportunity in structure determinations.1
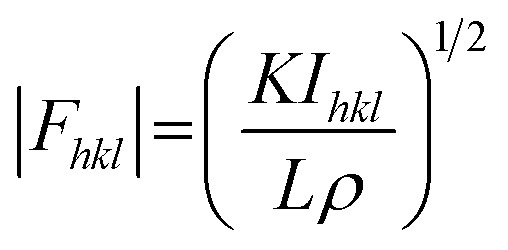
2
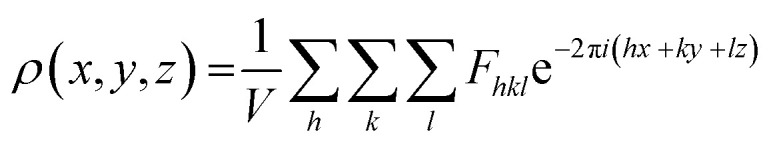
*L*: Lorentz factor, *ρ*: polarization factor, *K*: a constant for a given crystal in an experiment.

It is worth discussing the achievements of structure determinations by PXRD in MOFs—the foundational background for early accomplishments in solving the crystal structure of COFs using PXRD. Particularly, the *ab initio* method for determining structures of reticular materials was successfully accomplished for a family of Zr-based MOFs (UiO-66, -67, and -68) in 2008.^80^ Indeed, UiO-66 (1–2 μm crystal size) with high crystallinity showed diffraction peaks up to 100° 2*θ* (CuKα) corresponding to a resolution of *ca.* 1 Å. The crystal structure of UiO-66 was solved by the direct method implemented in the EXPO program; the steps of indexing, intensity extraction, and Rietveld refinement were carried out using the TOPAS program from Bruker. Especially, the Rietveld refinement factors including *R*_p_ and *R*_wp_ converge at very low values (0.016 for *R*_p_ and 0.022 for *R*_wp_) demonstrating the high agreement between the structural model and the experimental one. A similar method was used for elucidating the crystal structure of an exemplified Ti-based MOF, MIL-125.^81^

Rietveld refinement using high-resolution synchrotron PXRD patterns will effectively be applied to determine the crystal structures of crystalline materials. This technique allows us to refine the crystal structure at the atomic level even revealing the structural dynamics and host–guest interaction. Particularly, Zhang and co-workers reported the guest-dependent dynamics in COF-300 variants whose crystal structures were determined by synchrotron PXRD patterns.^82^ The authors were able to synthesize scalable COF-300 with high crystallinity. Interestingly, COF-300 exhibited structural dynamics which contracted upon taking up water molecules (6% reduction of unit cell volume). Additionally, upon inclusion of tetrahydrofuran (THF), the cell volume of COF-300 increased by 50%. The contraction and expansion were observed along with the changes of PXRD patterns and solved by Rietveld refinement with a very low reliability factor. This dynamic behavior of COF-300 was corroborated by water and THF uptake measurements, showing different steps in the adsorption isotherms.^82^ This work underlines the fundamental understanding of crystal structures of dynamic COFs which can be used for many applications relying on the flexible frameworks.

**Fig. 9:**
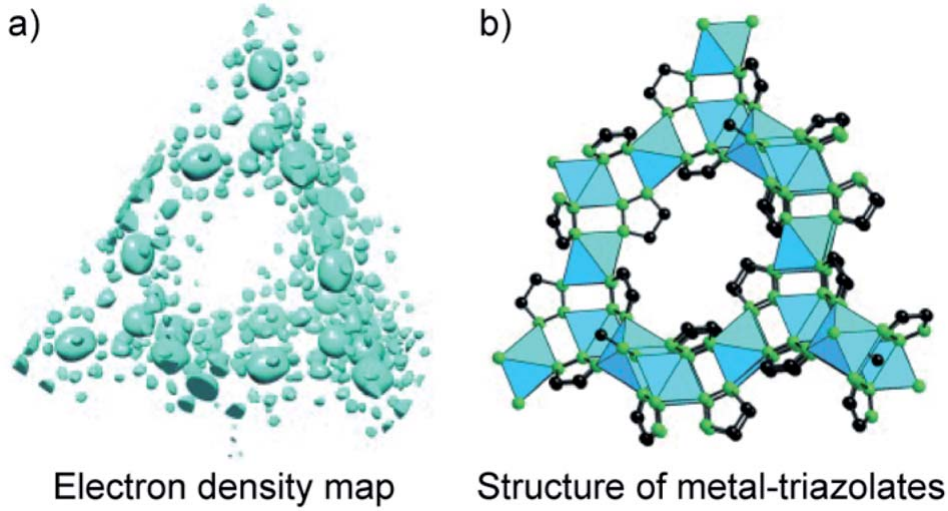
Charge-flipping method is applied to analyze the electron density map of metal-triazolates (a) represented in the form of structural connectivity (b). Reproduced with permission.^84^ Copyright 2012, Wiley-VCH.

Another method for structure determinations by PXRD is the charge-flipping method^83^ which is applied to solve MOF structures reported by Yaghi and co-workers in 2012 (Fig. 9).^84^ This method uses the dual-space algorithms, combining the direct-space modification with simple resubstitution of the experimental |*F*_*hkl*_| structure modulus. Importantly, the solution method is similar to that of single-crystal diffraction data and this further allows other modifications and/or iteration schemes (*e.g.*, histogram of chemical compositions).

In terms of crystallinity, COFs composed of solely organic building units are normally less crystalline than the MOF counterparts which means that solving the COF structure by PXRD is even more challenging. The structural elucidation of COFs, therefore, relies mainly on using the conceptual basis of the topological approach in light of reticular chemistry. This is understandable because most of the reported works have studied structures with predictable topologies based on predesigned SBUs (except for a few).^49^ However, as specified in Section 3 and well observed in MOF chemistry, novel topologies can be formed even when ‘usual’ geometrical structures of SBUs were employed (*i.e.*, such combinations normally form designed/predictable topologies). And if this is the case, without high crystallinity, it is likely impossible to solve the crystal structures. Especially, we were able to apply the charge-flipping method to solve the crystal structures of COF-432 (ref. 70) and COF-76 (ref. 85) whose structures consist of usual squares and triangles but they crystallize in **mtf** and ribbon-based topology, respectively. The topological approach of reticular chemistry in such cases does not work.

In the case of COF-432, the powder-pattern indexing resulted in unit cell parameters identical to the results from the ED data. In fact, the ED did not provide much information for COF-432 due to the low-resolution data. The electron density map (EDM) of COF-432, however, showed the fragments of 1,1,2,2-tetrakis(4-aminophenyl)ethene (ETTA) but not 1,3,5-triformylbenzene (TFB) building units. Such an EDM analysis suggested the locations of ETTA entities, thus reasonably allowing structure modeling of the material. It should be mentioned that we, unfortunately, could not perform the Rietveld refinement for COF-432 due to the fact that COF-432 may contain mobile molecular guests and defects which result in the deviation between the simulated PXRD pattern and the experimental one in terms of the relative intensity, as observed in the first few peaks.^70^

**Fig. 10:**
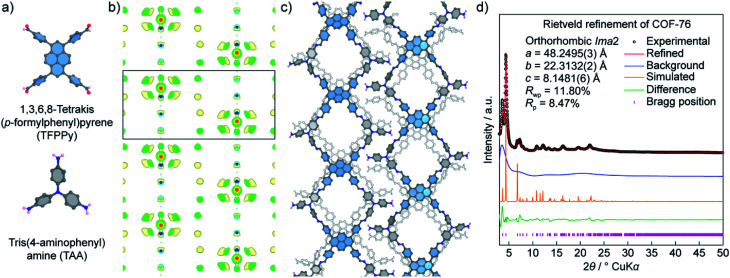
Structural analysis of COF-76 adopting a ribbon-like topology. (a) Chemical structures of square-planar {1,3,6,8-tetrakis(*p*-formylphenyl)pyrene (TFPPy)} and triangular {tris(4-aminophenyl)amine (TAA)} units used to form COF-76. (b) Electron density map analysis of COF-76 generated by the charge-flipping method displays the fragments of TFPPy arranged in the form of ribbons. (c) Crystal structure of COF-76 showing a ribbon-based topology. (d) Rietveld refinement of COF-76 demonstrates a good agreement between the experimental and simulated PXRD pattern.

Unlike COF-432, COF-76 is composed of 1D ribbons formed by substoichiometrically linking 1,3,6,8-tetrakis(*p*-formylphenyl)pyrene (TFPPy; square) and tris(4-aminophenyl)amine (TAA; triangle) thus generating a frustrated backbone. The EDM of COF-76 clearly indicates the high conjugation systems of square units (TFPPy) locating in parallel ribbons; the TFPPy entities between two ribbons in one layer are arranged in a zigzag manner (Fig. 10). Owing to the high crystallinity (*i.e.*, PXRD pattern shows more than 20 peaks), the COF-76 structure was refined with the Rietveld method leading to reasonable residual values of reliability profile factors (*R*_p_ = 0.0847, *R*_wp_ = 0.1180) (Fig. 10). These two studies underline the importance of using PXRD to solve the crystal structures of COFs, especially when a suitable crystal size cannot be obtained for the ED and SXRD analyses.

It should be noted that using PXRD to solve the crystal structures of crystalline materials in general and COFs in particular is facing great challenges because of the (1) low crystallinity of COFs, (2) peak overlapping issues, and (3) absence of heavy atoms (*i.e.*, low electron density). At this juncture, it is imperative to first obtain COFs with the highest crystallinity, and second to combine the structure determinations by PXRD with additional characterizations to validate the models, as presented comprehensively in the COF-432 case study. Nevertheless, it does not rule out the fact that the charge-flipping method, along with ED analysis, will be a powerful technique to aid the structure solution of COFs.

## 5. Conclusion

The chemistry of COFs encompasses the linking of discrete organic SBUs into extended structures that can be designed, predicted, and therefore precisely controlled using the conceptual basis of reticular chemistry. Manifested by the uncountable articles published in the past decade, well researched in over one hundred countries, COFs have proven to be one of the most interesting materials thanks to their advanced functional aspects—the exquisiteness of COFs in particular and reticular materials in general.

This article aims to provide as much knowledge of reticular design and crystallography in COF chemistry as possible and therefore it undoubtedly casts light on further understanding of the principle design, synthesis, and applications of COFs. We have deeply tapped into the reticular design of COFs to comprehensively overview the concept of reticular chemistry. Particularly, we have discussed (1) the design of COFs in rational ways that focus largely on the topological deconstruction, and (2) the method of modeling possible structures of targeted COFs, and highlighted the achievements of using this method to create novel topologies in 3D COFs. In order to educate (emerging) scholars on how the structures of COFs can be determined, we have finally presented the fundamentals and applications of 3D ED, SXRD, and PXRD techniques in solving the COF crystal structures. Particularly, the crystal structure determination of COFs starts with structural models which can be simply obtained by simulation using the foundational understanding of reticular chemistry. The simulation of COF structures works well with simple building units and those COF systems based on high symmetry. The models are subsequently corroborated and validated by further characterizations including Fourier-transform infrared spectroscopy, nuclear magnetic resonance spectroscopy, elemental analysis, thermogravimetric analysis, N_2_ or Ar sorption isotherms, and PXRD analysis. More importantly, N_2_ or Ar isotherms will provide informative pore size distributions of the model structures. We encourage emerging scholars to spend time to optimize the crystallinity of COFs so that advanced techniques (structural solution by PXRD and/or 3D ED) can be effectively used to elucidate COFs' crystal structures, which will be invaluable to study further applications of COFs. It should be noted that although the COF community has achieved some important results (*i.e.*, COFs with high crystallinity whose crystal structures can be fully determined by SXRD and/or 3D ED), COF chemistry needs an impactful revolution for crystallinity enhancement. Future research in COF chemistry—a young research field—may focus on enhancing crystal sizes, crystallinity, and discovering more novel topologies, which should be developed in parallel with the practical applications.

From the structural perspective, COFs with new topologies in many cases are formed serendipitously and do not follow the conceptual basis of reticular chemistry. This means ones who are willing to take risk and are curious about the novel chemistry of COFs will be looking forward more to such nature-reveals-itself products. Therefore, overcoming the crystallization limitation and comprehending structural determinations in COFs must be considered so that the chemistry of COFs would be diversified similar to their MOF counterparts.

## Author contributions

H. L. N. designed and directed the project and wrote the manuscript.

## Conflicts of interest

There are no conflicts to declare.
